# Management of Endodontic Flare-Up in the Presence of Periapical Radiolucency: Case Report and Overview

**DOI:** 10.7759/cureus.49719

**Published:** 2023-11-30

**Authors:** Amira Alghazaly, Lamees Al Habib

**Affiliations:** 1 Restorative Dental Sciences, Collage of Dentistry/Taibah University, Madinah, SAU; 2 Dentistry, Taibah University, Madinah, SAU

**Keywords:** endodontic management, peri-apical radiolucency, root canal treatment (rct), flare-up, clinical case report

## Abstract

Flare-ups following root canal therapy (RCT) are of significant concern in dentistry. They are characterized by the occurrence of pain or swelling during or even after a root canal procedure. They affect a considerable proportion of cases, up to 16% and occasionally even 50%, and they often necessitate unscheduled appointments. Whenever a flare-up occurs within hours or days following RCT, it demands emergency treatment. Flare-ups can be attributed to multiple factors, including mechanical, chemical, and microbial causes. Identifying these factors beforehand and utilizing the expertise of clinicians can help better manage patients after the procedure and assure a long-term successful outcome. It has been observed that retreatment cases with periapical periodontitis treated in a single appointment have higher flare-up rates. Despite the prevalence and the impact of flare-ups, the endodontic community has yet to adopt scientifically validated and approved preventive measures to manage and prevent flare-ups. Hence, in this case report, the ethical letter was approved (TUCDREC/250923). It provides insight into the management of an interappointment flare-up with an overview.

## Introduction

Root canal therapy (RCT) aims to eliminate or at least entomb bacteria from an infected root canal [1]. Encountering a flare-up after an RCT appointment presents a serious concern [2]. Within the domain of endodontics, the term “flare-up” is commonly used to describe the occurrence of notable pain and/or swelling that follows RCT [2]. This discomfort can be significant to the extent that it calls for an unscheduled visit [2]. One study estimated that between 3% and 50% of patients who undergo RCT will experience postoperative pain [3]. After an RCT, flare-ups can occur as soon as a few hours later or as late as a few days later [4]. Studies have shown that flare-ups are likely to occur after endodontic therapy as much as 16% of the time and even up to 50% in some circumstances [5]. Several factors may lead to periapical inflammation, including mechanical factors such as instrumentation (hand files or rotary), chemicals such as medicament that are introduced into the periapical area, or the extrusion of debris at the apex [6]. These factors depend mainly on the type of tooth, root canal system configuration, periapical lesions status, sinus tract existence, tooth vitality, and intracanal medications [7]. In contrast, one study found no association between flare-ups and age, gender, or different arch or tooth groups [8]. The ability to predict the occurrence of flare-ups during the perioperative period and utilize the experience of clinicians can greatly enhance postoperative patient management when appropriately recognized [9]. Flare-ups were most frequently attributed to pulpal necrosis without periapical pathosis (6%), followed by cases where pulpal necrosis coexisted with periapical pathosis (52%), which was one of the more frequent causes of flare-ups. [2]. Flare-ups were also observed in situations involving irreversible pulpitis (22%) [2]. Considering the number of treatment visits, a higher frequency of flare-ups was observed in teeth that received multiple visits compared to those that had a single visit [2]. Furthermore, studies have elucidated that individuals with periapical lesions have a heightened risk of experiencing pain and flare-up episodes in contrast to their counterparts lacking these pathological conditions [2]. Upon the development of this damage, the body’s defensive system initiates a fighting response, resulting in swelling and pain [4]. Accordingly, the intensity of flare-ups depends mainly on the quantity and virulence of microorganisms in the periodontal tissues [3]. In contrast, the treatment will depend on whether it is a primary endodontic treatment or a retreatment with intracanal medication [10]. If there is a high level of bone destruction appearing in the radiograph, there is a high risk of post-endodontic flare-up; for cases where bone destruction exceeds 5 mm, there is a greater likelihood of experiencing pain [11]. It is common practice in modern dentistry to provide nonsurgical endodontic treatment [12]. RCT has saved millions of teeth by revolutionizing material science and techniques [12]. The advancements in surgical, prosthetic, and restorative care have reduced the difficulty of tooth replacement, but saving a natural tooth with a good outlook remains the best option compared to having a tooth extracted and replaced [13]. RCT involves removing the filling material from the canal and then cleaning, shaping, and sealing the canal [14]. For nonsurgical retreatments to successfully reestablish healthy periapical tissues and achieve predictable results, gutta-percha must be completely removed from root canal walls to reestablish working length (WL), disinfection must be promoted, and the canals must be re-obturated [15]. Endodontic hand files, nickel-titanium rotary instruments, Gates Glidden burs, heated instruments, ultrasonic instruments, lasers, and adjunctive solvents have all been proposed as methods of removing filling materials from root canal systems [16]. Traditionally, the removal of gutta-percha, whether with or without the use of solvents, can be a laborious and time-consuming procedure, particularly when the filling material is densely compacted [16].

## Case presentation

**Figure 1:**
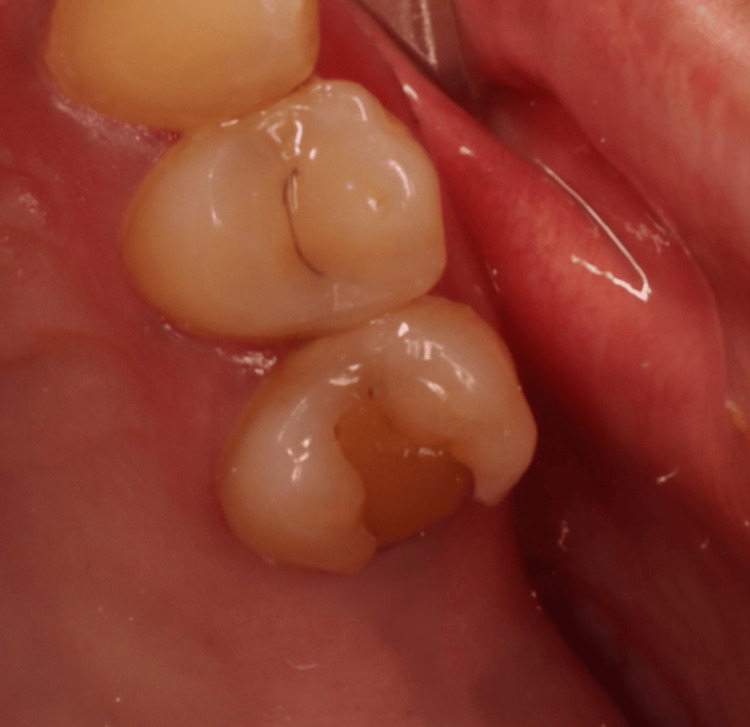
Intraoral occlusal photograph shows that #25 has discolored broken restoration with a large gap and recurrent caries around the restoration.

**Figure 2:**
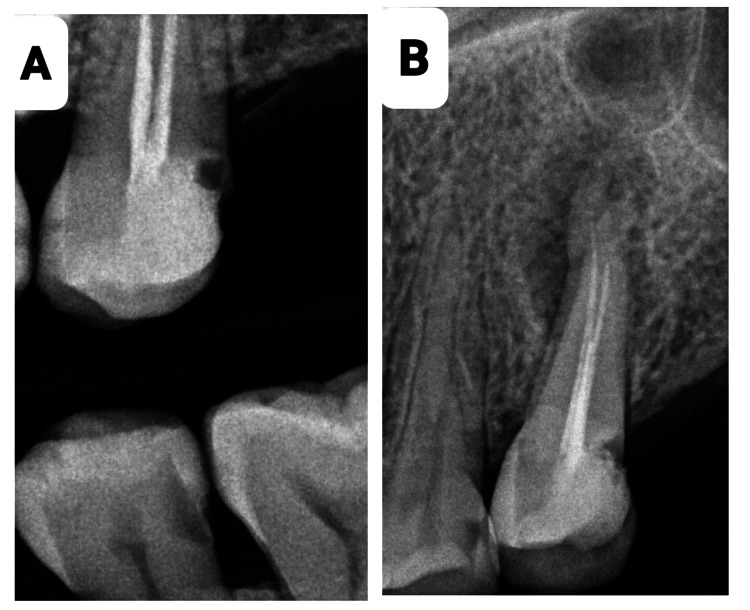
Intraoral bitewing (A) and periapical (B) radiographs show recurrent caries under the restoration and a previous RCT with short obturation and large radiolucency at the root apex.

**Figure 3:**
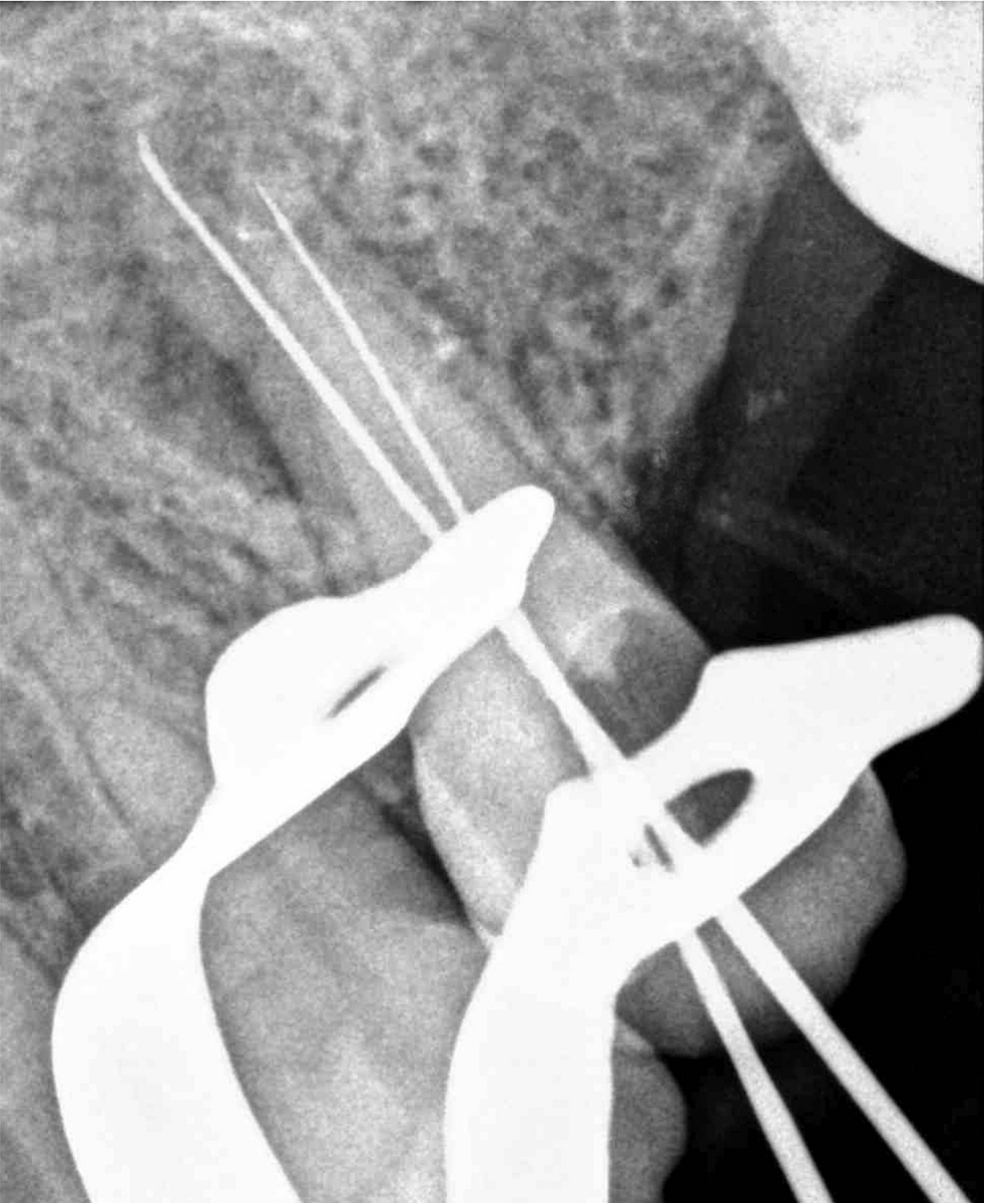
Working length was determined using an apex locator and confirmed with an intraoral periapical radiograph.

**Figure 4:**
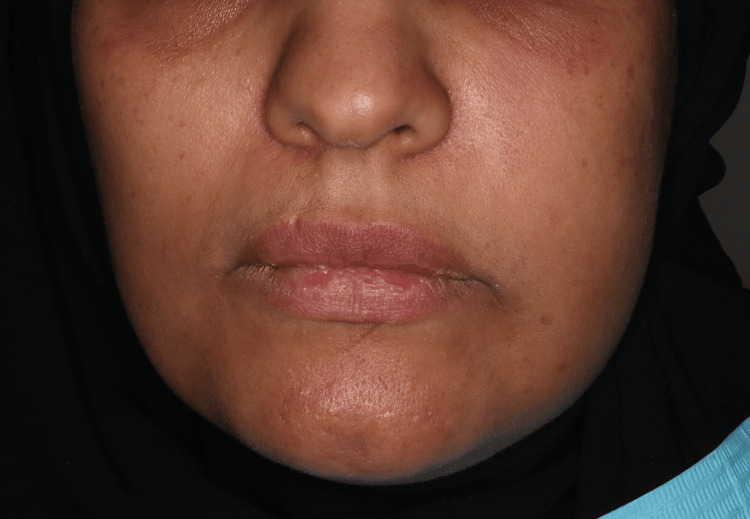
Extra-oral photograph shows swelling on the left side of the face, extending to her left cheek and slightly involving the lower eyelid.

**Figure 5:**
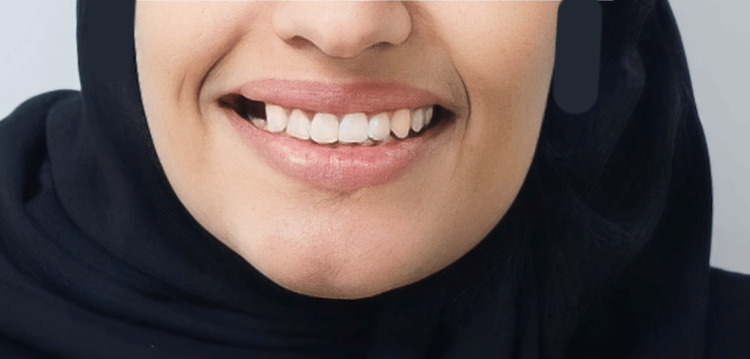
Extra-oral photograph shows that the swelling had subsided completely after seven days.

**Figure 6:**
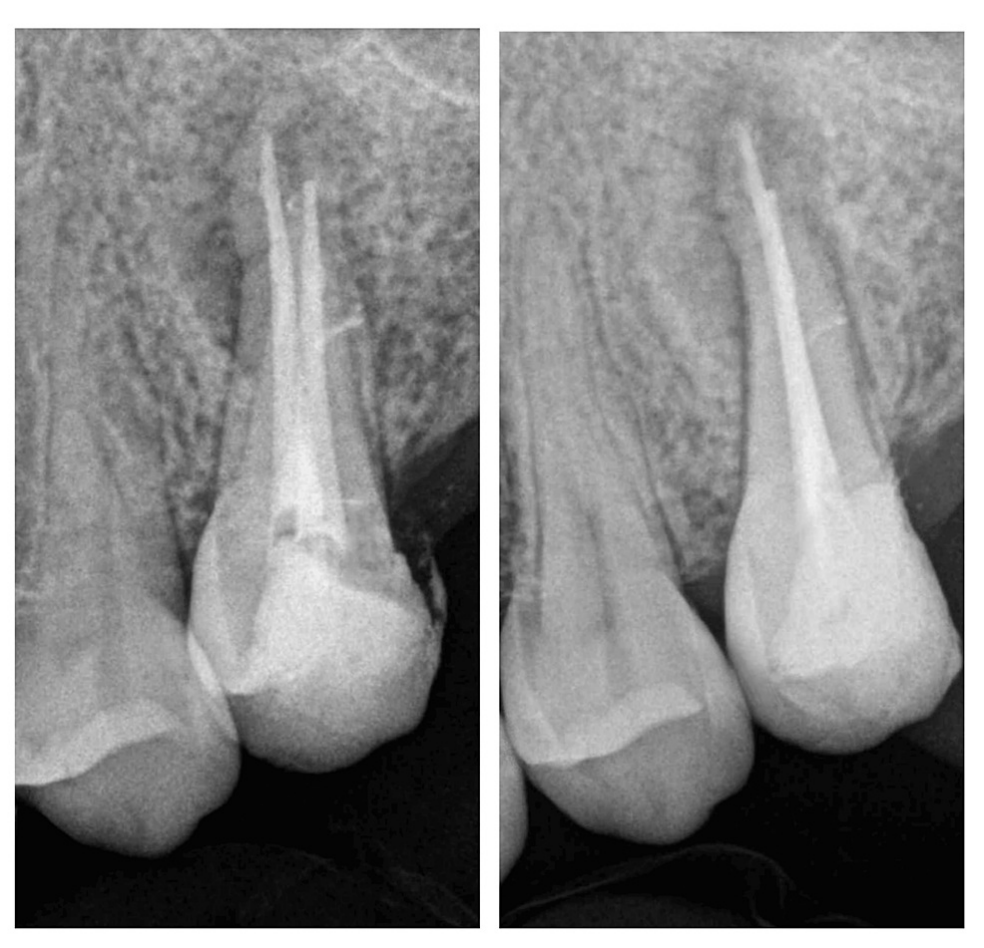
Intraoral periapical radiograph shows obturation of the root canal of tooth #25.

**Figure 7:**
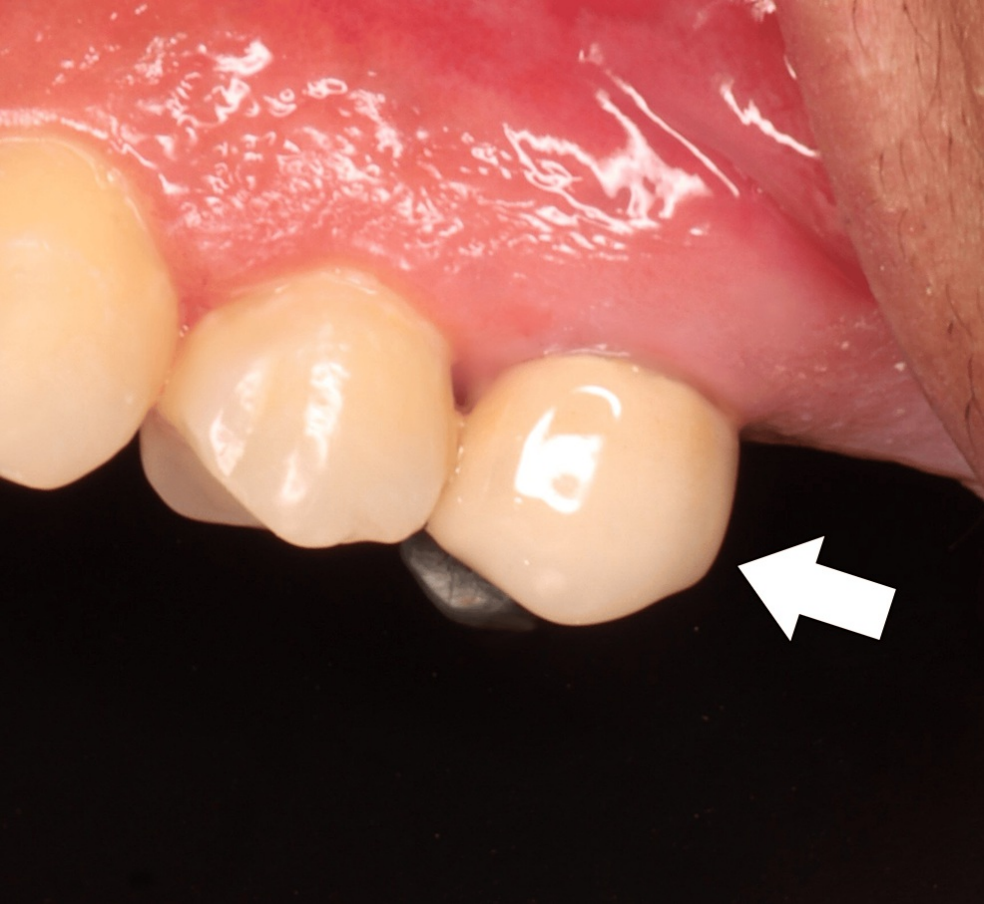
Intraoral photograph shows a full-coverage crown (PFM) done on tooth #25.

A 43-year-old female patient was admitted to the dental clinic of Taibah University with the complaint of severe pain from tooth #25 with no significant findings regarding medical or social history. However, the patient gave a history of previous RCTs related to this tooth. Intraoral examination revealed a discolored, broken restoration with a large gap and recurrent caries under the restoration (Figure 1). Tooth #25 was tender to percussion with normal mobility. It was slightly sensitive to palpation, and no deep pockets were found related to this tooth. Intraoral, periapical, and bitewing radiographs were done, which showed recurrent caries under the restoration, along with a previous RCT with short obturation quality and large radiolucency related to the root apex (Figure 2). The concluding diagnosis was previously treated with an acute apical abscess. After discussing this with the patient, the treatment decision was to do an RCT. After giving local anesthesia to the patient, the old restoration and the caries were excavated under rubber dam isolation and removed, then the access cavity was troughed to the correct outline with complete deroofing, and root canals were located. Gutta-percha was removed from the coronal part first using Gates Glidden sizes 3, 2, and 1, then the gutta-percha was dissolved with the use of H files and solvent. Canal patency was regained. WL was determined using an apex locator and confirmed with an intraoral digital periapical radiograph (Figure 3). Biomechanical preparation was completed using hand instruments with a step-back technique under copious irrigation with 5.25% sodium hypochlorite solution followed by 2 mL of saline. The first appointment was finished by placing calcium hydroxide-based intra-canal medicament; a closed dressing of zinc oxide eugenol temporary restoration was placed in the access cavity; and occlusion checking was done. Two days after the first appointment, the patient was contacted and reported severe pain and swelling on the left side of the face, extending to her left cheek, and slightly involving the lower eyelid (Figure 4). To alleviate the patient’s concerns, the problem was explained, and she was advised to use cold packs for 15 minutes on, then 15 minutes off, followed by a warm pack to help alleviate and reduce the swelling. In the emergency appointment for the flare-up, the patient reported that the swelling had diminished compared to the previous night following the application of both an ice pack and a warm pack. The temporary restoration was removed under rubber dam isolation, and the canals were irrigated with copious amounts of normal saline. Occlusal contact was confirmed using the articulating paper. Using a diamond bur in a high-speed handpiece with copious water spray, all occlusal contacts were reduced on the cusps and marginal ridges to relieve the pain associated with biting. Antibiotics and analgesics were prescribed for the patient for five days. The patient was contacted daily until the next appointment, at which point all signs and symptoms subsided. After seven days, the swelling had subsided completely (Figure 5), and the patient became asymptomatic. Obturation of the root canal was completed using cold lateral compaction of the gutta-percha (Figure 6). The access cavity was subsequently restored with composite resin. The patient was advised to have a full-coverage crown. After following up for three weeks, the full coverage crown was done on tooth #25 (Figure 7). The patient was followed up with for four more weeks after crown cementation.

## Discussion

The target of endodontic treatment is to eradicate harmful microorganisms, but this can be difficult to accomplish due to the complex nature of the root canal system [17]. When dealing with occasions when the endodontic treatment fails, nonsurgical endodontic retreatment is typically preferred if access to the root canals is feasible [18]. In the literature, several studies have found that around 45% of endodontically treated teeth still exhibit persistent apical periodontitis, which is primarily caused by infection after microorganisms survive the initial treatment of both intra- and extraradiculars of the root canal system [18]. In cases where periradicular lesions are absent, it is expected that endodontic treatment will have a positive outcome [18]. However, there can be considerable variation in success rates if lesions are present, ranging from 31.8% to 85% [18]. Thus, the presence of bacteria is generally attributed to treatment failure [18]. This is primarily caused by inadequate chemomechanical debridement and improper filling of the canals, which fail to provide a proper seal at the apex [19].

One important factor in considering the microbial cause as an etiological cause is recurrent caries, a new microbial entry in an area where coronal restoration has already been performed but not properly sealed. If left in place for more than two weeks between appointments or after treatment, a delay in applying the final restoration can be a potential factor for flare-ups [5]. The presence of bacteria in the root canal system can lead to the reestablishment of microorganisms in the root canal system, and the subsequent migration of bacteria and debris to surrounding tissues can result in inflammation and pain [5]. Hence, the major concern is the apico-coronal seal; when disturbed, microorganisms can thrive and multiply, leading to the development or continuation of a periradicular lesion [19]. Bacteria and host defense mechanisms are generally in balance with each other [4]. Acute periradicular inflammation occurs when this balance is disturbed in favor of microbial aggression [4]. During the procedure, debris can be inadvertently extruded, the root canal microflora can change, or the environment can affect the procedure [4]. Incomplete chemical and mechanical preparations, secondary intraradicular infections, and increased oxidation-reduction potential within the root canal can all cause facultative bacteria to grow [4].

There are several factors that affect the development of pain associated with endodontic infections, including pathogenic strains, virulent clonal types, the number of cells, and microbial interactions [4]. Some studies have suggested that peri-radicular diseases are associated more with certain species of bacteria. Porphyromonas species are more associated with symptomatic peri-radicular lesions and abscessed teeth [20], and Prevotella and Peptostreptococcus species cause acute clinical symptoms [21]. The most common bacteria causing pain with percussion were Peptostreptococcus, Eubacterium, Porphyromonas endodontalis, P. gingivalis, and Prevotella species [22]. Several studies have suggested that Gram-negative anaerobic bacteria tend to cause chronic endodontic infections, including acute abscesses [20].

Treatment failure is caused by all the factors discussed previously when flare-ups occur [22]. After or during endodontic treatment, patients experience intense pain and swelling in the oral mucosa and facial tissues [23]. Following root canal procedures, this condition typically starts within hours or days and is severe enough to demand an unscheduled appointment [23]. In addition to the factors mentioned above, the severity of the periapical lesion and irritation of that area also contribute to flare-ups [5]. Various elements should be considered in this regard, such as the number of treatment sessions required, the type of intracanal medicaments used, any pain experienced before the procedure associated with the periapical region, the diagnosis of the pulp and periarticular region, and whether the treatment is an initial or retreatment [2]. The ongoing debate in endodontics concerns the decision of whether to perform RCT in one visit or multiple visits [2]. One study showed that patients may experience postoperative pain after the root canal filling in single-visit therapy [1]. However, single-visit therapy offers the advantage of eliminating the need for a temporary seal and potential leakage between appointments [1]. It also reduces the time spent in the dental chair and the number of visits for the patient, leading to higher acceptance of this approach [1]. Some studies have shown that flare-ups and pain are more common after single visits, whereas others indicate that they are more common after multiple visits [1]. One possible explanation for this difference is the possibility that necrotic tissue, which is more likely to cause periapical inflammation and microbial colonization, can be pushed out during cleaning and shaping in a single-visit procedure, leading to inflammation [1]. It has been found that patients who did not receive irrigation of their root canals exhibited greater frequency and levels of pain than those who received irrigation with 5.25% sodium hypochlorite and 3% hydrogen peroxide [2]. However, sodium hypochlorite (NaOCl), although possessing strong antimicrobial properties, can cause significant toxicity if it extrudes into the periradicular tissues [2]. Therefore, apical extrusion should be prevented during irrigation so that pain between appointments will be experienced less frequently [2].

Endodontic treatments involving chemicals such as intracanal medicaments, irrigation solutions, and sealers may have the potential to be toxic and result in irritation and flare-ups if they come into contact with the periradicular tissues [5]. The magnitude of the inflammatory response will vary depending on the quantity of substances extruded [5]. A 2010 study identified that irrigation with 5.25% sodium hypochlorite was associated with greater pain compared to irrigation with a 2% chlorhexidine solution [5]. Another examination of flare-ups and their relationship to the initial diagnosis was conducted within the context of endodontic therapy [2]. Flare-ups are significantly predicted by the presence of periapical lesions in necrotic teeth [2]. According to multiple studies, periapical lesions are associated with more pain and flare-ups than those without such lesions [2]. Furthermore, the enlargement of the apical foramen during therapy is associated with a higher level of debris extrusion and greater movement of microorganisms and irrigative solutions into the periodontal tissues, causing inflammation [5]. Accordingly, flare-ups can be influenced by the size of the apical foramen [5].

The use of variable endodontic kinematics and techniques directly addresses the problem of flare-ups [24]. Reciprocation instruments result in more debris extrusion apically; hence, more inflammation was found [24]. Notably, a study conducted by Reddy and Hicks revealed that, compared to cleaning with NiTi rotational mechanical instruments, manual instruments employing the step-back technique extrude more debris into the periradicular tissues [5]. These insights provide valuable information for improving endodontic treatment strategies and minimizing the occurrence of flare-ups [5]. Moreover, precise determination of the WL is crucial in endodontic treatment to avoid flare-ups, which can result from incorrect WL estimation [5]. Overestimating the WL can also lead to over-instrumentation, causing the extrusion of infected debris and filling material into the periradicular tissues; this extrusion can cause irritation and inflammation [5]. Determining the cause of the flare-ups will facilitate the maneuver [25].

Unfortunately, the exact causes of flare-ups are not always known, as highlighted by Shenoy et al. [25]. Consequently, treatment options have been proposed, such as occlusal relief before endodontic therapy to prevent postoperative pain [25]. In conjunction with localized treatment approaches, including re-instrumentation, placement of intracanal medicaments, and drainage establishment, they are employed to manage flare-ups [25] because they lead to improved outcomes and fewer postoperative complications [25]. In the current case, the flare-up was determined to require nonsurgical retreatment to manage postoperative treatment complications. Interappointment emergencies after root canal retreatment were found to be higher than the initial RCT [7]. The demonstration of a higher flare-up incidence compared to initial treatment underlines the significance of selecting a technique that minimizes postoperative discomfort [9,11]. One study found that if endodontic retreatment is performed when clinical symptoms relate to periradicular changes, chemical preparation and filling of the root canal can be completed in a single visit [26]. However, if there are radiological changes in the periradicular tissues, to achieve maximum root canal disinfection, it is best to make two visits [26]. Flare-up rates are nearly five times higher after a single endodontic retreatment compared with retreatment over two visits [26]. The swelling should be treated with cold compresses at first to stimulate local microcirculation, and after one day, warm compresses and warm mouth rinses should be used [27].

To treat post-treatment pain definitively, the access cavity of the symptomatic tooth needs to be reopened [28]. The procedure continues with thorough debridement and copious irrigation to remove remaining tissue, microorganisms, and toxic products, as symptoms of post-treatment are largely caused by these factors [28]. Post-endodontic pain can be effectively controlled with irrigation with normal saline at room temperature or colder [29]. In addition, it may be an effective alternative to analgesics [29]. One study found that intracanal steroids or corticosteroid antibiotic compounds placed after root canal debridement are effective in reducing post-treatment pain [30]. Multiple studies have shown that antibiotics used prophylactically do not impact flare-up incidence [5]. Postoperative pain has been reported to be reduced by using analgesics and anti-inflammatory drugs before treatment [5]. Combining non-steroidal anti-inflammatory agents (NSAIDs) and opiates is effective in flare-ups [5]. Rather than prophylactically administering antibiotics before RCT, this is considered therapeutic rather than preventative [31]. In addition, necrotic pulps display periapical radiolucencies on radiographic examinations [31]. It is inevitable that such radiolucencies are infected and that antibiotics need to be used therapeutically [31]. Some researchers have concluded that amoxicillin antibiotics should not be given before undergoing endodontic treatment [31]. For the purpose of treating any existing infection, systemic antibiotics should not be prescribed unless there is a clear indication that antibiotics are necessary [31]. Moreover, it has been shown that occlusal reduction reduces postoperative pain in teeth that causes pain when biting [32]. Consequently, sensitized nociceptors are alleviated by mechanical stimulation [32]. Whereas Rosenberg et al. found that occlusal reduction reduces postoperative pain [33], multiple studies did not show a significant difference in postoperative pain between RCTs with and without occlusal reduction [34]. This discrepancy highlights the need for further investigation into the effectiveness of occlusal reduction in RCT.

## Conclusions

The management of flare-ups in endodontic treatment is crucial to achieving successful results. It is possible to experience flare-ups due to factors including pre-existing infections, intraoperative mishaps, real-time monitoring, and previous treatments. There are meticulous techniques that can help minimize the risk of flare-ups. A patient’s education and postoperative care are essential for preventing and managing any potential complications. A collaborative approach between the dental practitioner and patient is crucial for the successful completion of endodontic treatment as well as minimizing the incidence of flare-ups. In recent studies, some steps or guidelines have been shown to reduce its incidence. Flare-up incidence is influenced by many factors, and a specific treatment protocol is important for management and prevention. The establishment of such a procedure will require further studies.
